# Resilience and preparedness of hospitals for pandemics: Lessons learned from COVID-19

**DOI:** 10.4102/jamba.v16i2.1804

**Published:** 2024-10-16

**Authors:** Tlou D. Raphela

**Affiliations:** 1 Department of Disaster Management Training and Education Centre for Africa, Faculty of Natural and Agricultural Sciences, University of the Free State, Bloemfontein, South Africa

**Keywords:** resilience, vulnerability, COVID-19, preparedness, health care centres

## Abstract

Amidst the COVID-19 pandemic, health systems around the world took a lot of pressure, and the resilience of these systems has been addressed in thedisaster management and COVID-19 literature. However, little focus has been given to the resilience of hospitals in general. Therefore, the study assessed one hospital in the Free State province of South Africa as a preliminary study for a project that will look at all hospitals in the Free State province. The study assessed the following: (1) the preparedness and actionable strategies that were implemented across the waves of the COVID-19 pandemic; (2) the resilience plans of the hospitals and also determined (3) the resilience of the hospital amid the COVID-19 pandemic; (4) the vulnerabilities exposed by the COVID-19 pandemic in the hospital. Using the R statistical program and modelling, I found that the study hospital was not prepared holistically for the COVID-19 pandemic. The five generalised linear mixed models and the general linear models applied did not show significant differences for the most important variables used to measure resilience. The models showed the hospital to be less resilient to pandemics. Several vulnerabilities were recorded across the hospital.

**Contribution:**

This study’s findings suggest that the studied hospital is not resilient to pandemics and will be able to make recommendations to relevant government departments to work together to strengthen the resilience of the country’s healthcare system once the project is completed.

## Introduction

Amid the coronavirus disease 2019 (COVID-19) pandemic, countries recognised the possibility of additional surges, and hospitals were supposed to examine why some facilities were able to sustain effective operations while others struggled. However, that was not the case in most developing countries as healthcare workers and hospital management were more focussed on COVID-19 patients. Harris et al. (2021) reported traditional approaches to understanding hospital pandemic responses to focus on the ‘4S’ framework of preparedness: staff, stuff, space and systems. A lot of hospitals struggled with COVID-19 surges despite plenty of lead time and extensive resources to implement the 4S framework (Kadri et al. 2021). It is now clear that attention to these factors was necessary but not sufficient simply because COVID-19 is novel. A complementary approach is to understand the pandemic response through the lens of organisational resilience. The idea that resilience matters, and that studying variation in past resilience can inform policy that promotes future resilience, is a promising way to examine hospitals’ responses to the pandemic (Barbash & Kahn 2021).

This study, therefore, set out to evaluate the resilience of hospitals during the COVID-19 pandemic: A case of one hospital in the Free State province, South Africa.

## Research methods and design

The research design is a systematic plan and procedure to be followed in integrating the different components of the study coherently and logically to effectively address the research problem and answer the research questions (Leedy & Ormrod 2015). This study adopted a mixed-method research design to obtain qualitative and quantitative data. The quantitative component of the study was used to collect numeric data and the qualitative component was used to collect text data (Dubois 2018). A mixed-methods study, therefore, allows for a more holistic research approach (Leedy & Ormrod 2015).

### Target population and sampling

This study reports preliminary results from one hospital in the Mangaung Metropolitan Municipality. The study is part of a bigger project that awaits funding that targeted 30 hospitals across the Free State province. The sample size for this study included 60 healthcare workers from one of the 30 hospitals across the province. This study targeted Pelonomi Hospital in Bloemfontein as a case study because this hospital has been reported to have a lot of issues that were exacerbated by COVID-19 (Ross 2020). Pelonomi Hospital is a regional large size hospital that represents one of the previously disadvantaged health institutions in the Free State (Daffue, Joubert & Otto 2016). Also, Pelonomi has been experiencing high levels of industrial action in the past years (Daffue et al. 2016). The study opted for an in-depth case study of this hospital rather than focussing on many institutions.

The major target groups in this study were the Pelonomi Hospital management and health workers across the hospital. A stratified proportional random sample (in terms of job description) was drawn from the different categories of healthcare workers. The study used a sampling in the form of a computer printout of all workers in the hospital obtained from the Pelonomi Hospital’s personnel division. Randon sampling of the respondents from each job category was from a population of 745 and 8% of the population was sampled which gave a sample size of 60. The population of healthcare workers at Pelonomi Hospital is relatively large; only those who are directly involved in healthcare were interviewed. The study used published tables to determine the sample size in addition to the 8% selection criteria to gather the opinions of the healthcare workers.

### Data collection and analysis

I used only a semi-questionnaire survey to collect data for this study. The questionnaire was piloted with 10 healthcare workers before the administration to check for validity and reliability. For the pilot study, a preliminary analysis of the collected data was done to check the reliability and validity of the questionnaire. A representative sample of 10 participants from the target population for the main study was selected to ensure that the findings from the pilot study applied to the larger study. The questionnaire was administered to the 10 participants to test for content, predictive, construct and concurrent validity (Leedy & Ormrod 1980). I compared the pilot study results with those from established instruments measuring the same construct. For predictive validity, this pilot study made it possible for the author to set up future studies. For reliability, internal consistency was used with the questionnaire data collected from the 10 participants, and Cronbach’s alpha was used to measure how well the items in the questionnaire measured the same construct and a figure of more than 70% was found for the questions that were tested making the questionnaire valid; there were no changes that were made on the questionnaire after piloting.

The questionnaire used for this study was developed by the researchers. Data were captured on an Excel spreadsheet and converted to a CSV (comma-separated value) file before it was imported into *R* Statistical Software (version 4.2.0; 2022-04-22) for statistical analysis while text analysis was done for the qualitative part of the study to derive themes that were surfacing from the gathered data.

### Study questions

This study set out to answer the following four main questions.

1. What would organisational resilience look like in the context of COVID-19?
2. How were COVID-19 surges responded to in ways that preserve standards of care for patients without COVID-19 in your hospital?
3. How did your hospital preserve access to care for the entire community of patients you serve?
4. Was there adequate provision of PPC or PPE and clear communication from hospital management and the government to make the healthcare workers feel valued amid the COVID-19 pandemic?

### Statistical analysis

I ran a series of Generalised Linear Mixed Models (GLMM) with a Poisson distribution to answer main questions one to three and ran one Generalised Linear Model (GLZ) with a binomial distribution to analyse question four of this study. All models were run using the *R* statistical software (*R* version 4.2.0 (2022-04-22) and the models were two-tailed with alpha set at 0.05. The study reported coefficient estimates, including the residual degrees of freedom, standard errors, *Z*-values, corresponding *p*-values for the GLZ and the Wald statistics, and *p*-values from the analysis of variance (ANOVA) of the model for all GLMMs applied across the study. All graphs were produced using the ggplot2 package from the *R* statistical software.

### Organisational resilience in the context of COVID-19

To answer the first question of the study, I demonstrated what would organisational resilience look like in the context of COVID-19 by applying five separate GLMMs and setting the number of healthcare workers’ reports as the response variable for all four models as follows:

Model 1 – to assess how the hospital responds to a surge in COVID-19 cases, I set up the responses (ensure the delivery of high-quality, moderate-quality and poor-quality care for patients) and the dichotomous ‘yes’ and ‘no’ responses as the independent variables.

Model 2 – To identify where patients with COVID-19 were admitted in the study hospital, I set the responses (well-staffed, moderately staffed and poor-staffed units) and the dichotomous ‘yes’ and ‘no’ responses as the independent variables.

Model 3 – To ascertain whether patients with COVID-19 in the study hospital were handled by doctors and healthcare staff who have the skills and experience to provide appropriate care, I set up the responses (always, most times and sometimes) and the dichotomous ‘yes’ and ‘no’ responses as the independent variables. As a follow-up question, I asked if the hospital has units that are specific for highly infectious diseases.

Model 4 – In this last model on organisational resilience, I ran the GLMM on a follow-up question that assessed whether the study hospital coordinated with other regional hospitals and transport services to transfer patients rapidly and safely to capable centres. I set up the responses (always, most times, and sometimes) and the dichotomous ‘yes’ and ‘no’ responses as the independent variables for this model.

### Preservation standards of care for patients without COVID-19

To address question 2 of the study, I demonstrated how COVID-19 surges were responded to in ways that preserve standards of care for patients without COVID-19. I set the number of healthcare workers’ reports as the response variable and three questions related to emergency patients’ needs and the healthcare workers responses (always, sometimes, not at all) to those questions as the independent variable. These three questions were analysed together using one GLMM with a Poisson distribution even though they were asked separately in the study questionnaire.

### Hospital preservation of access to care for the entire community

To answer question 3 of the study, I assessed how the study hospital preserved access to care for the entire community of patients they serve. I set the number of healthcare workers’ responses (count data) as the dependent variable and responses (continuation with elective surgeries and mitigating the exacerbation of health disparities amid the COVID-19 pandemic) as independent variables. I applied one GLMM with a Poisson distribution for both questions as the responses were the same (always, sometimes and not at all).

### Hospital provision of personal protective equipment, personal protective clothing and adequate communication

Question 4 of the study was addressed by applying a GLZ model. The questions about the provision of PPC, PPE and the question that asked the healthcare workers if there was clear communication from hospital management or the government to make them feel valued amid the COVID-19 pandemic were analysed together using a GLZ, with a binomial distribution and logit-link function. The model is comprised of first-order effects and interaction effects between the following variables: responses, answers and the ‘yes’ and ‘no’ responses for the responses and answers as the dependent variable. Tukey’s honestly significant difference (Tukey’s HSD) post hoc test was used to obtain pairwise level of significance categorical predictors of data (*N* = 60).

For all GLMM applied across the study, the number of respondents was set as the random factor. The GLMM and GLZ statistical analyses were chosen to analyse data for this study because this study aimed to apply robust statistics that will assess relationships between variables to answer the questions of this study.

### Ethical considerations

Approval to conduct the study was obtained from the University of the Free State Human Ethics Committee, (No. UFS-HSD2022/0854/22).

## Results

### Organisational resilience in the context of COVID-19

The results of a GLMM showed healthcare workers’ responses when asked how their hospital responded to a surge in COVID-19 cases (Wald $\chi_{2}^{2}$ = 1.51; *p* = 0.468), dichotomous responses of ‘yes’ and ‘no’ (Wald $\chi_{1}^{2}$ = 0.00; *p* = 0.995) were not significant predictors of the number of the health care workers’ report. In addition, healthcare workers’ responses when asked where patients with COVID-19 were admitted to their hospital (Wald $\chi_{1}^{2}$ = 1.01; *p* = 0.600) did not predict the number of healthcare workers’ reports. However, the dichotomous responses of ‘yes’ and ‘no’ (Wald $\chi_{1}^{2}$ = 12.29; *p* = 0.000) when that question was asked was a significant predictor of the number of healthcare workers’ reports.

**FIGURE 1:**
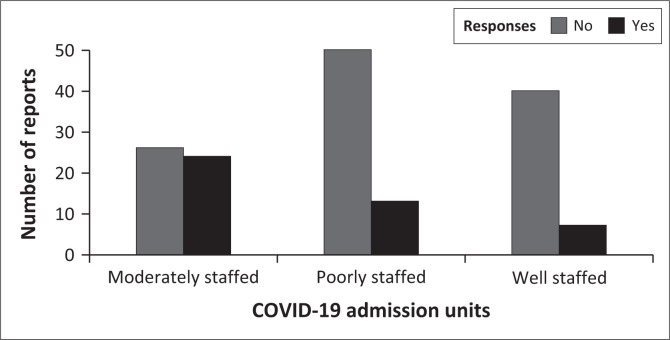
Healthcare workers’ reports on COVID-19 patients admission in their hospital.

Moreover, the number of healthcare workers who reported ‘no’ when asked if patients with COVID-19 were admitted in moderately, poorly and well-staffed units was the highest across the study (Figure 1). However, the negligible number of reports when healthcare workers were asked if patients with COVID-19 were admitted to well-staffed units staffed units shown by our study (Figure 1) was worrisome, considering that COVID-19 was dubbed the deadliest disease by the World Health Organization (WHO) in 2021.

The results of a GLMM showed that the responses when healthcare workers were asked whether patients with COVID-19 in their hospital were handled by doctors and healthcare staff who have skills and experience to provide appropriate care (Wald $\chi_{2}^{2}$ = 0.40; *p* = 0.818), and the dichotomous responses of ‘yes’ and ‘no’ (Wald $\chi_{1}^{2}$ = 3.54; *p* = 0.059) were not significant predictors of the number of health care workers reports. In addition, the highest number (*n* = 40) of healthcare workers reported that the hospital does not have units that are specific for highly infectious diseases.

**FIGURE 2:**
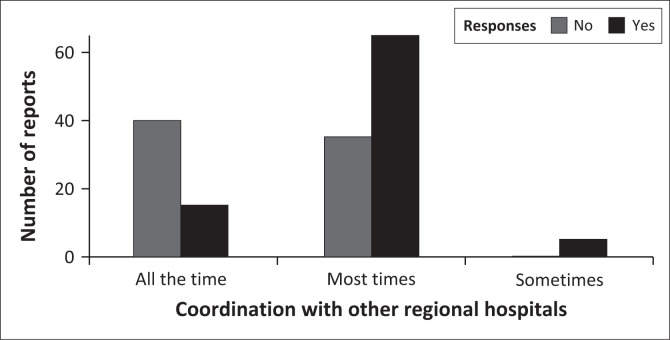
Healthcare workers’ reports on coordination with other regional hospitals and transport services to transfer patients amid the COVID-19 pandemic.

Furthermore, when the healthcare workers were asked whether their hospital coordinated with other regional hospitals and transport services to transfer patients rapidly and safely to capable centres, the GLMM model showed a statistical significance (Wald $\chi_{2}^{2}$ = 23.37; *p* < 0.001) for the responses to the number of reports by healthcare workers, but dichotomous responses of ‘yes’ and ‘no’ (Wald $\chi_{1}^{2}$ = 0.18; *p* = 0.667) did not predict the number of healthcare workers reports. Moreover, the majority of the respondents reported ‘yes’ when asked whether their hospital coordinated with other regional hospitals and transport services to transfer patients rapidly and safely to capable centres (Figure 2). However, the majority of the healthcare workers reporting ‘no’ to all the time (Figure 2) shows less resilient hospitals when it comes to collaborations.

### Preservation standards of care for patients without COVID-19

**FIGURE 3:**
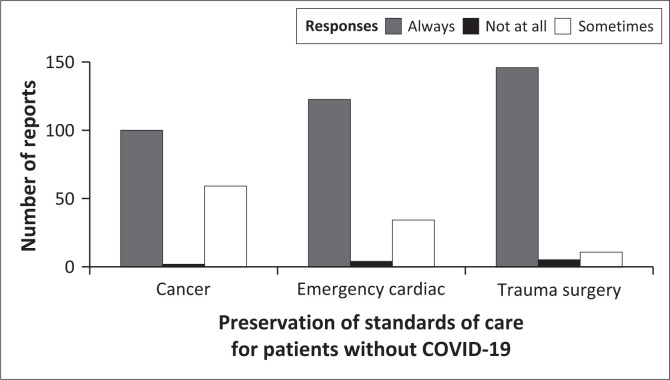
Healthcare workers’ reports about the preservation of standards of care for patients without COVID-19.

The results of a GLMM showed that responses when healthcare workers were asked if patients who needed cancer treatment, emergency cardiac care and trauma surgery (asked separately) were given priority amid the COVID-19 pandemic (Wald $\chi_{2}^{2}$ = 65.23; *p* < 0.001) was a significant predictor of the number of reports, but the responses to those questions (always, sometimes and not at all) (Wald $\chi_{2}^{2}$ = 0.67; *p* = 0.714) did not predict the number of the healthcare workers reports. Furthermore, among the three emergency care needs, cancer patients were the most neglected as the least number of healthcare workers reported that always (Figure 3).

### Hospital preservation of access to care for the entire community

**FIGURE 4:**
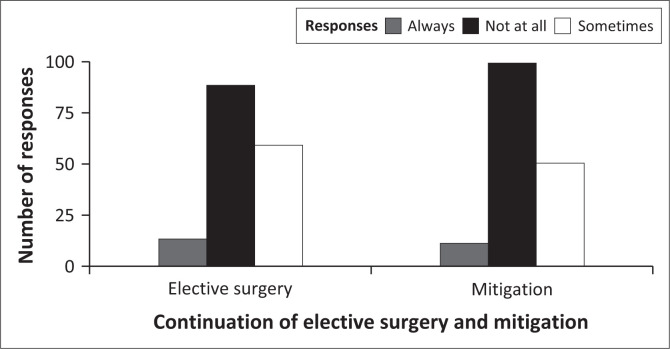
Healthcare workers’ reports about the continuation of elective surgery and mitigating the exacerbation of health disparities amid the COVID-19 pandemic.

The results of a GLMM showed that the number of health workers’ reports was significantly predicted by the responses when the healthcare workers were asked about the continuation of elective surgery and mitigating the exacerbation of health disparities amid the COVID-19 pandemic (Wald $\chi_{2}^{2}$ = 96.39; *p* < 0.001). However, the number of healthcare workers’ responses for the trichotomous variable with three levels always, sometimes, and not at all were not significant predictors of the number of responses (Wald $\chi_{1}^{2}$ = 0.67; *p* = 0.714). A very negligible number of healthcare workers reported a continuation of elective surgery and mitigating the exacerbation of health disparities amid the COVID-19 pandemic (Figure 4).

### Hospital provision of personal protective equipment, personal protective clothing and adequate communication

The ANOVA of the binomial distribution GLZ model showed that responses when the healthcare workers were asked about the adequacy of the provision of PPC and PPE and whether there were clear communications from hospital management or the government to make them feel valued amid the COVID-19 pandemic were significant predictors of the dichotomous yes and no responses ($\chi_{3}^{2}$ = 34.71, *p* < 0.001). However, the answers given (always, sometimes, not at all) for that response ($\chi_{2}^{2}$ = 0.00, *p* = 0.99) and the interaction between the responses and the answers ($\chi_{2}^{2}$ = 1.45, *p* = 0.48) did not predict the yes and no responses as the binomial outcome.

**FIGURE 5:**
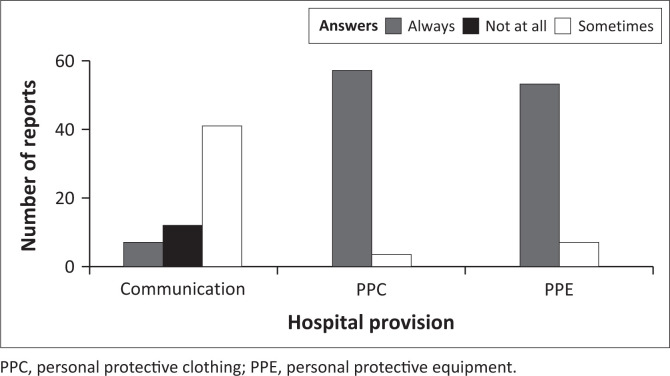
Healthcare workers reports on hospital provisions amid the COVID-19 pandemic.

Furthermore, the highest responses across the study were from healthcare workers who reported that they were provided with PPC always (Figure 5). Interestingly, the highest number of healthcare workers reported that there were sometimes communications between hospital management and the government to make them feel valued amid the COVID-19 pandemic (Figure 1). Moreover, significant differences were found for responses to PPE and PPC but not for communication (Table 1).

**TABLE 1:** Output of a Generalised Linear Model showing healthcare workers’ reports about the provisions of personal protective equipment, personal protective clothing and communication adequacy amid the COVID-19 pandemic.

Coefficients	s.e.	*Z*	*P*
Responses: Communication	−0.916	−1.09	0.273
Responses: PPC	**0.277**	**−2.21**	**0.026**
Responses: PPE	0.383	−4.50	**< 0.001**
Answer: Not at all	1.140	−0.60	0.543
Answer: Sometimes	0.946	−0.89	0.370
Responses: PPC - Answer: Not at all	NA	NA	NA
Responses: PPE - Answer: Not at all	NA	NA	NA
Responses: PPC - Answer: Sometimes	0.769	1.57	0.624
Responses: PPE - Answer: Sometimes	1.658	1.32	0.209

PPC, personal protective clothing; PPE, personal protective equipment; s.e., standard error; NA, not applicable.

Note: Two coefficients are not defined because of singularity, significant figures are shown in bold.

## Discussion

### Organisational resilience in the context of COVID-19

Firstly, resilient hospitals would respond to a surge in COVID-19 cases in ways that ensure the delivery of high-quality care for patients with the disease. Secondly, patients with COVID-19 would be admitted to well-staffed specific units with doctors and other healthcare personnel who have the skills and experience necessary to provide appropriate care. If such units were not available, resilient hospitals would coordinate with regional hospitals and transport services to transfer these patients rapidly and safely to capable centres (Barbash & Kahn 2021). However, in the study hospital that was not the case. There were reports of make-shift triages in parking lots in most government hospitals across South Africa (Crowley et al. 2021; Kuguyo, Kengne & Dandara 2020).

### Preservation standards of care for patients without COVID-19

Resilient hospitals would respond to COVID-19 surges in ways that preserve standards of care for patients without COVID-19, such as those needing cancer care, emergency cardiac care and trauma surgery. Hospital managers would acknowledge that although trade-offs exist, mitigating the unintended effects of surge responses by accommodating urgent needs of non–COVID–19 patients is as important as addressing the needs of patients with COVID-19 (Barbash & Kahn 2021). Again, there were several reports of neglect of chronic patients across South African public hospitals in the early stages of COVID-19 (Dashraath et al. 2020).

### Hospital preservation of access to care for the entire community

Naturally resilient hospitals would preserve access to care for the entire community of patients they serve, continuing elective surgeries and mitigating the exacerbation of health disparities during the pandemic. This was done on an ad hoc basis in the study hospital as more focus was on the COVID-19 patients.

### Hospital provision of personal protective equipment, personal protective clothing and adequate communication

Because COVID-19 is novel, the resilience of the study hospital differs from traditional conceptions of preparedness for disasters such as evacuation plans in case of flooding, structural fires, load-shedding and communication failure. Therefore, a hospital that was prepared only for the Zika virus outbreak in 2015 would not have been prepared for COVID-19. Preparations for the Zika virus-focussed on prenatal clinical and laboratory support (Madad et al. 2018). However, COVID-19 presented hospitals with challenges of PPEs and PPC in addition to the much-needed moral support for healthcare workers. Consistent with this study’s findings, other healthcare workers reported that they were sometimes provided with adequate PPE and PPC (Figure 5) amid the reported deadliest COVID-19 pandemic (Friedman & Akre 2021). Preparedness for COVID-19, such as having a large stockpile of ventilators and N95 masks, was not a possibility for South Africa as a developing country, and this availability was to translate to a resilient hospital.

This study results revealed the highest number of healthcare workers who reported that there were sometimes communications from hospital management and the government to make them feel valued amid the COVID-19 pandemic. This shows the gap that needs to be bridged between taking care of patients by healthcare workers and appreciation or appraisal policies in South Africa for frontline workers.

## Conclusion

In conclusion, a resilient hospital will continue with elective surgery and mitigation of exacerbating health disparities, while also protecting the well-being of their staff, not just by ensuring adequate PPE and PPC but also through clear communication from leaders that make staff feel valued and connected to the organisational mission. There were complaints of inadequate PPE and PPCs across hospitals in South Africa (De Filippis et al. 2022). Moreover, studies across the world have reported how frontline workers’ well-being was affected by the COVID-19 pandemic (Chew et al. 2020; Gavin et al. 2020; Kinman, Teoh & Harriss 2020).

The factors that create resilient hospitals remain poorly understood, and a more nuanced understanding of what it means to be a resilient hospital will provide novel strategies to create resilience ahead of the next pandemic. COVID-19 will not be the last large-scale public health threat of the 21st century. In addition to infectious diseases, hospitals and health systems will confront climate-mediated extreme weather events, cyberterrorism disruptions and other threats in the decades to come. Hospitals can never be truly prepared for these events. However, if hospitals understand and build sustainable resilience, they will be ready.

The study findings highlighted complaints of inadequate PPEs and PPC across hospitals in South Africa, underscoring a significant area for improvement (De Filippis et al. 2022). Furthermore, studies have consistently reported the adverse impact of the COVID-19 pandemic on frontline workers’ well-being globally (Chew et al. 2020; Gavin et al. 2020; Kinman et al. 2020), emphasising the need for robust support systems within healthcare settings (Barbash & Kahn 2021).

### Recommendations

In this context, hospitals should resist the temptation to simply prepare for the next pandemic only by creating the infrastructure and procedures they lacked during COVID-19. Efforts to stockpile supplies and increase intensive care unit (ICU) bed capacity will be useful if the next pandemic is like COVID-19 but would also be redundant and inefficient, increasing healthcare costs without improving day-to-day quality.

Instead, hospitals should implement changes that will be of value no matter the challenges they may encounter and that will be useful even during normal times. Among these changes may be more robust supply chains, cultures of excellence and collaboration, and systems for coordinating operations within and across hospitals. At the same time, the health services research enterprise should conduct rigorous studies investigating which organisational elements are most important for fostering hospital resilience (Barbash & Kahn 2021).

The factors contributing to hospital resilience remain inadequately understood. A more nuanced understanding of hospital resilience is crucial for developing novel strategies to enhance preparedness for future pandemics. As this study indicates, resilience involves multifaceted approaches, including maintaining elective surgeries, ensuring equity in healthcare delivery and supporting staff well-being through adequate resources and effective leadership communication. Given that COVID-19 will not be the last large-scale public health threat of the 21st century and considering other emerging threats such as climate-mediated extreme weather events, hospitals must build sustainable resilience to be ready for these challenges. While complete preparedness for all potential events is unattainable, hospitals that understand and implement principles of sustainable resilience will be better equipped to respond effectively.
